# Can surgery follow the dictates of the pandemic “keep your distance”? Requirements with COVID-19 for hygiene, resources and the team

**DOI:** 10.3205/dgkh000354

**Published:** 2020-08-03

**Authors:** Colin M. Krüger, Axel Kramer, Andreas Türler, Hartwig Riediger

**Affiliations:** 1 Immanuel Klinikum Rüdersdorf, Abt. Chirurgie, Zentrum für Robotik, Rüdersdorf b. Berlin, Germany; 2 Institut für Hygiene und Umweltmedizin, Universitätsmedizin Greifswald, Greifswald, Germany; 3 Johanniter Kliniken Bonn, Abteilung für Viszeralchirurgie, Bonn, Germany; 4 Vivantes Humboldt Klinikum, Department für Chirurgie, Berlin, Germany

**Keywords:** COVID 19, infection prevention, surgical team, personal protective equipment, ventilation, surgery procedure, insufflation, robotics

## Abstract

Since the beginning of the pandemic, there have been restrictions in the daily care of surgical patients – both elective and emergency. Readying supply capacities and establishing isolation areas and areas for suspected cases in the clinics have led to keeping beds free for treating (suspected) COVID-19 cases. It was therefore necessary to temporarily postpone elective surgery. Now, elective care can be gradually resumed with the second phase of the pandemic in Germany. However, it remains the order of the day to adapt pre-, intra- and post-operative procedures to the new COVID-19 conditions while maintaining specialized hygiene measures. This concerns the correct procedure for the use of personal protective materials as well as process adjustment for parallel treatment of positive and negative patients in the central OR, and handling of aerosols in the operating theater, operating room, and surgical site under consideration of staff and patient protection. Although dealing with surgical smoke in the operating theater has long been criticized, COVID-19 is forcing a renaissance in this area. Finally, the choice of surgical method, whether open surgery or minimally invasive procedures, is critical in determining how many colleagues are exposed to the risk of infection from COVID-19 patients, sometimes for hours. Here, robot-assisted surgery can comply with the pandemic’s requirement to “keep your distance” in a unique way, since the surgeon can operate at virtually any distance from the surgical site, at least with regard to aerosol formation and exposure.

## Zusammenfassung

Mit Beginn der Pandemie hat es Einschränkungen in der täglichen Versorgung chirurgischer Patienten – elektiv ebenso wie im Notfall – gegeben. Bereitstellung von Versorgungskapazitäten und die Einrichtung von Isolations- und Verdachtsbereichen in den Kliniken haben dazu geführt, Bettenkapazitäten freizustellen. Damit war es erforderlich, temporär Eingriffe der elektiven Versorgung zurückzustellen. Mit Eintreten in die zweite Phase der Pandemie in Deutschland kann schrittweise die Elektivversorgung wieder aufgenommen werden. Es bleibt jedoch Gebot der Stunde, unter Vorhaltung spezialisierter Hygienemaßnahmen die chirurgischen Abläufe prä-, intra- und postoperativ an die Gegebenheiten unter COVID-19 anzupassen. Das betrifft den Ablauf bei der Verwendung persönlicher Schutzausrüstung wie auch die Prozessanpassung bei der parallelen Versorgung COVID-19-positiver und -negativer Patienten in einem Zentral-OP, den Umgang mit Aerosolen im OP-Trakt, im OP-Saal und am OP-Situs unter Berücksichtigung des Schutzes der Mitarbeiterinnen und Mitarbeiter wie auch der Patienten im OP. Steht der Umgang mit chirurgischen Rauchgasen im OP bereits lange in der Kritik, bekommt das unter COVID-19 eine Renaissance mit Nachdruck. Nicht zuletzt die Wahl der OP-Methode, offen chirurgisch oder minimal invasiv, entscheidet, wieviele Kollegen am an COVID-19-Erkrankten zum Teil über Stunden einem Infektionsrisiko ausgesetzt sind. Hier kann die robotisch assistierte Chirurgie dem Gebot der Pandemie „Abstand halten“ in besonderer Weise Folge leisten, da der Chirurg während des Eingriffs quasi beliebig weit vom OP-Situs, zumindest in Bezug auf Aerosolbildung und Exposition, entfernt seine Arbeit tut.

## Introduction

With the beginning of the COVID-19 pandemic, fundamental changes have taken place in medical care in Germany and globally. The preventive, containment, and medical requirements are fundamentally the same, but are being implemented to varying degrees due to different resources of individual countries. Since the outbreak of the pandemic in November 2019 in China, the understanding of preventive measures has been steadily growing. The German healthcare system has had time to draw conclusions from the findings in China, but also elsewhere in Europe, especially Italy and Spain. 

There were two central factors in the German hospital landscape that led to the restrictions described below: First, the call by the German Federal Ministry of Health to substantially increase the number of intensive care beds which would allow invasive ventilation of patients by temporarily postponing elective procedures; and second, the shortage of personal protective equipment (PPE), which is mainly produced in China, that accompanied the beginning of the pandemic.

While the problem of PPE procurement was centrally organized by the federal and state governments, each hospital had to secure intensive care resources by reassigning or recruiting personnel to adequately staff isolation areas and ICUs. Part of the new intensive care personnel to be recruited came from the surgical staff, which consequently immediately reduced daily operating-theater capacity. In addition, inpatient capacities had to be reallocated to create COVID-19 isolation and pre-isolation areas to protect the wards.

For the surgical department, this meant the immediate suspension of elective surgical procedures. In the recommendations of 24 April 2020, the DGAV (German Society of General and Visceral Surgery) compiled a list of diseases that could be considered as indications for urgent surgery [1]. The Federal Ministry of Health has not yet issued a uniform, binding and nationwide guideline for the surgical departments.

Since then, the following needs have arisen for the surgical clinics:

1. Definition of the range of surgical procedures to be continued
2. Creation and management of patient waiting lists
3. Surgical patient care with reduced intensive care and inpatient bed capacity
4. Establishment of care structures for COVID-19 patients (including suspected cases) in the operating theater
5. Maintaining training and education in the pandemic situation

The definition of the range of interventions to be continued has recently been amended. The Federal Minister of Health, with his publication of the fact paper on the New Daily Routine for Hospitals of 27 April 2020 [2], cleared the way for the resumption elective surgery (see no. 1, above). The administration of patient lists generated to date will thus be highly influenced by the regional implementation of this regulation by the states, as well as by the expected renewed increase in the number of newly infected and sick patients after the relaxation of protective measures (see no. 2, above). 

A bottleneck in the near future will be the intensive care capacity for elective surgery patients. The newly created intensive care capacities will be generated in particular by the nursing staff reassigned from peripheral care areas, which will be able to help out in the intensive care area if necessary after intensive training in recent weeks. Since we were already confronted with the much-discussed shortage of nurses in Germany before the pandemic, the clinics have been forced to reduce the number of inpatient beds or, if necessary, to carry out short-term personnel rescheduling. This does not increase planning security for patients who require intensive medical monitoring and care in the early post-operative phase (see no. 3, above). 

If the establishment of a care structure for COVID-19 patients (Pa-COV19) is successful, it would facilitate the rapid return to a well-organized operative care. Comparable to the establishment of isolation and pre-isolation areas in the ward block, this requires parallel structures in the operating theater in order to safely care for Pa-COV19 and guarantee human and material resources for the duration of the pandemic.

## Room requirements in the operating area

The spatial and building technology situation is usually set. Because very few clinics have spatially separate surgical units for Pa-COV19 and non-Pa-COV19, the analysis below begins with the inward transfer into the operating theatre and ends with the outward transfer.

The hygiene requirements of the pandemic regulation in Germany mean that central areas of the OR tract, such as induction areas and recovery rooms, must not be used simultaneously by Pa-COV19 and non-Pa-COV19. As a result, in addition to its primary function, the operating theatre should also be used for the induction of surgery and for the phase of early post-operative monitoring. The path of Pa-COV19 in the OR tract is thus reduced to the operating room with direct insertion. Anaesthesia preparation, induction and discharge are performed in the closed OR. The operating room functions as a recovery room for the patient. Postoperatively, the Pa-COV19 is transferred to the isolation area of the intensive care unit or directly to the pre-ISO ward in suspected cases or to the isolation ward if SARS-CoV-2 is detected.

## Surgical hygiene and ventilation

SARS-CoV-2 is transmitted by droplet infection. Aerosols from infected carriers pose a particular risk. However, aerogenic spread also takes place. The problem is that testing by deep throat swabbing during the incubation period (2–14 days) [3] may be negative, although the carrier is already infectious. As a result of replication in the throat area, the virus thus also reaches the upper and lower gastrointestinal tract, which means that fluids from these areas can also be considered infectious during surgery. Suspected cases of COVID-19 and confirmed positive patients must be treated equally in the operating theatre. The hazard for the staff in the OR is defined by contact with patient-related aerosols. 

In accordance with the regulations of DIN 1946-4:2018-09 (Room Air Technology – Part 4 [4], Tab. 1 Item 5.1: Infectious patients), the following ventilation requirements exist for the space in the intensive care unit: staff and third parties must be protected from infectious patients (e.g., patients with multi-resistant tuberculosis). Here, the technical requirements for room air requirements apply: patient rooms with supply and exhaust air and negative air balance to the airlock; airlock with negative air balance to adjacent corridors. As a result, these ventilation requirements must be implemented in the pre-, peri-, intra- and post-operative treatment process for infectious patients in whom aerosol formation is to be expected during treatment.

Ideally, the room air-conditioning system (RATS) in the operating theater can be switched to negative pressure. This ensures that no viruses from the OR are able to escape into neighboring rooms. Since opened doors immediately interrupt the negative pressure, air is exchanged with the environment during door opening. Therefore, the doors must be kept closed during surgery. When switching to negative pressure, it is recommended that the surgical field be flushed antiseptically before the surgical suture is applied, in order to kill pathogens originating from the room air and entering the surgical field, due to potential turbulence. With antiseptic irrigation, a reduction of postoperative wound infections can be achieved even without this additional risk factor [5].

In ORs that do not allow negative pressure maintenance, the overflow technique contaminates neighbouring rooms. Although contamination is lower due to the considerably higher ventilation flow in class 1a (LAF, laminar air flow) operating theaters than in air from mixed-ventilation operating theaters (class 1b). However, since SARS-CoV-2 can survive in room air as an aerosol for 16 hours [6], there is a risk of infection during this period. Operating theaters with LAF have a considerably larger ventilation volume flow than operating theaters with mixed ventilation (1b), which means that the aerosol dilution in the operating theatre with LAF is considerably faster. In operating theatres with LAF, the directional rather than merely mixing ventilation in the OR area also ensures additional protection for the surgical team and the patient. Due to the characteristics described above, operating theaters of room class Ib are associated with a higher risk of contamination for the OR team. It is questionable whether the FFP3 mask guarantees such a tight seal that the team is not endangered. In this case, secure protection of the surgical team can be achieved with overpressure body-exhaust suits [7]. With LAF, the surgical team is protected; however, due to the approximately 80-fold air change/h, adjacent rooms are contaminated with overflow technology. If, however, the air should flow directly out of the operating room, the operating theater can be used. The PPE described would be sufficient.

We have created a simple control protocol for ventilation evaluation and validated it in the flue gas video test. Compared to the OR standard ventilation, the RATS protocol for the OR is adapted as follows:

1. Supply air is reduced from 360 Pa 200 Pa.
2. Exhaust air is increased from 180 Pa 300 Pa.
3. A negative flow of 100 Pa results when the Laminar Flow Ceiling remains active.

Operating theaters or ORs of room class II with RATS without sterile filters are not appropriate, for the same reason as operating theaters with turbulent mixed flow. 

Operating theaters without an HVAC are also out of the question, since there is no dilution of aerosols released and the highest aerosol concentration occurs after opening the door at the end of the operating theater.

## Protective materials and personnel

**Figure 1:**
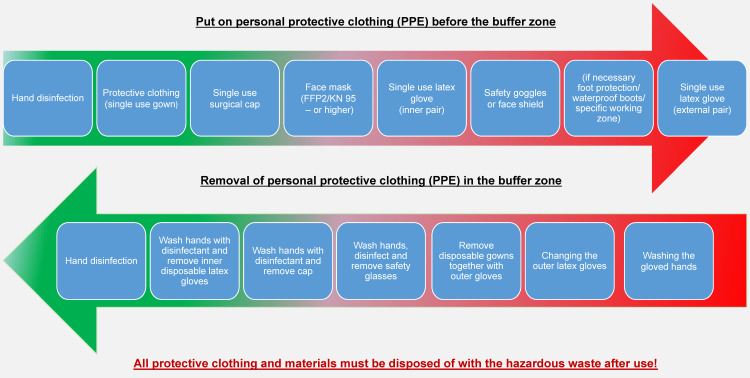
Correct process flow of inward and outward transfer into the isolation or infection area

In the recommendation of the RKI (Robert Koch Institute) on hygiene measures for the treatment and care of patients with a SARS-CoV-2 infection as of 24 April 2020 [8], Paragraph B, supplementary measures in the clinical field/personal protection measures/personal protective equipment comprise use of PPE consisting of protective gown, disposable gloves, at least tightly fitting mouth-and-nose or respiratory mask and safety goggles. In the direct care of patients with confirmed or probable COVID-19, at least FFP2 masks and 2 pairs of gloves must be worn in accordance with the occupational safety regulations [9]. With the Ebola outbreak, the importance of correctly putting on and taking off the PPE became obvious, in order to prevent infection when the PPE is taken off. It is recommended that staff be trained by the hygiene team to put on and take off the PPE according to a standardized trained procedure (Figure 1 (Fig. 1)), which was successful established in the University Medicine as well.

Particular attention should be paid to all activities that may be associated with aerosol formation (e.g., intubation or bronchoscopy). This means that in case of danger (suspected and confirmed COVID-19 infection), everyone in the operating room must be equipped with an FFP3 mask, but at least with an FFP2 mask. The protective materials are to be used on a patient-specific basis and are to be changed from patient to patient. In the event of supply bottlenecks, the measures for reuse of protective masks described in TRBA 250 and ABAS Decision 609 in the event of a pandemic can be helpful [9], [10]. 

### PPE in the OR procedures

Due to the transmission of SARS-CoV-2 by aerosols from the respiratory tract, respiratory and other surgery-related aerosols must be avoided or protective measures taken to prevent their transmission to staff and patients. According to the recommendations of the RKI [8], at least one FFP2 mask should be worn in direct patient contact in the case of justified suspicion and confirmed infection with COVID-19. Depending on material availability, this means one FFP3 mask for daily routine in the anesthesia/high risk/intubation department, and for all others in the operating theater at least one FFP2 mask. The recommendations of the DGAV from 24 April 2020 [1] suggest a mouth and nose protective mask for the rest of the OR team and an FFP2 mask for the anesthesia team, which in the authors’ view does not correspond to the strict interpretation of the current RKI recommendations. 

Uncertainty exists with regard to the surgically produced aerosols from mono- and bipolar cutting of tissue [11] as well as the aerosol generation during minimally invasive surgery, which are generated in the course of insufflation. No information is currently available on the infectivity of aerosol from pleural and/or peritoneal fluid. However, it is certain that viruses are detectable in the lungs and upper and lower gastrointestinal tract [12]. Fecal or oral transmission is therefore not excluded, but has not yet been proven [13]. For laparoscopy and pleural minimally invasive procedures, there is at least a theoretical risk of infectious aerosols in dissecting and resecting procedures on the lungs as well as the gastrointestinal tract. In addition, SARS-CoV2 is detectable in the blood at a frequency of 15%, which must be taken into account when bloody aerosols are formed (e.g., in vascular corrosion or orthopedic/accident surgery). As the role of the vapors from electric cautery has not yet been clarified, this should either be avoided or an additional smoke extraction system should be used. 

The choice of surgical procedure should continue to be based on the principle of “primum nihil nocere”. Thus, the best possible procedure currently clinically established for the treatment of a disease with the least invasiveness for the patient should be chosen. 

### Personnel

The personnel in the OR is to be reduced to the necessary minimum, optimally to:

1. surgeon + 1 assistant
2. anaesthesiologist + 1 anaesthesia nurse; the work of the circulating nurse in the OR is delegated to the anaesthesia nurse; the circulating nurse communicates by telephone with the room team for any additional material requirements
3. OTA (physician‘s assistant)

Since the risk of exposure to patient-related aerosols is considered to be highest during in- and extubation, but also during surgery directly on the patient, “keep your distance” is to be taken as given, even during the ongoing surgical procedure for everyone who is able to do so, i.e., operate at a distance. 

## Insufflation

In accordance with physiological specifications in the pressure structure of the venous vascular system, insufflation pressures of 12–15 mmHg have been established as the standard in laparoscopy [14], [15], [16]. Lower pressures of 8–10 mmHg are recommended in children and patients with premature cardiopulmonary disease and, in some studies, have been found to be superior to mechanical retraction systems [17], [18], [19]. Insufflators of the current generation can produce these low intracavitary target pressures with good intraoperative performance. Trocar sites should be kept tight by using assisting sutures or suitable trocar systems [20], [21], [22]. 

Modern two-lumen insufflation systems with “smoke evacuation” function and dissipative smoke filtration are preferable to others. Some of these systems also include the function of directed desufflation towards the end of the operating theater. Alternatively, older generations of insufflators with an established disposable smoke evacuation filter (according to DIN EN1822-1:2019) [23], [24] with a Luer-Lock connection can be used to render filtered smoke evacuation. 

Before intubation, as a pre-exposure prophylaxis, it is recommended that the oral cavity be irrigated with 1.25% aqueous PVP-iodine solution, if possible in combination with gargling. The patient is asked to rinse the oral cavity thoroughly, spit out the solution, and then gargle with fresh solution. Contraindications are hyperthyroidism, autonomous adenoma of the thyroid gland, and very rarely

## Surgery procedure

### Open surgery

There are voices – unfortunately without citable references – which proclaim the return to open surgery under COVID-19 circumstances with the argumentation of less aerosol production and quicker surgery. Open surgery is more personnel-intensive and requires 2 to 3, occasionally even 4 medical colleagues plus instrumental OTA over the patient for the duration of surgery. The advantage is the isobaric setting in the operating field, although tissue-specific aerosols can also be generated in the operating field during electrocoagulation.

### Conventional laparoscopy

In the opinion of the DGAV, there is nothing fundamentally wrong with performing laparoscopy in accordance with the published recommendations of 24 April 2020, provided that the protective measures mentioned above are implemented. One advantage may be the reduced number of surgeons, which is limited to the surgeon and camera assistant in the vast majority of laparoscopic procedures. Also, the involvement of the OTA is usually less than in open surgery.

### Robot-assisted surgery

In the past 5 years, robot-assisted surgery has established itself worldwide as a special form of laparoscopy, also in visceral surgery. Currently still far from being considered a “gold standard”, the evaluation is undergoing a change based on the first randomized studies of this technique comparing laparoscopy vs. robotics [25] in terms of oncological precision, reduced intraoperative blood loss, shortened inpatient intensive care stay and shortened hospital stay in various indications. While the previous path of robot-assisted surgery was often rocky, not least from an economic point of view, robot-assisted surgery obviously conforms to “keeping your distance” from the pandemic perspective. No other surgical technique in visceral and thoracic surgery is able to reduce the number of high-risk surgeons on patients in a comparable way.

This applies to simple operations such as hernias on the groin or diaphragm, up to complex operations on the pancreas, stomach, esophagus and the colorectum. The “first assistant” in the operating field is occasionally needed to change instruments, to apply a suture or a compress at the situs. As a rule, the surgeon can perform the operation alone from the console, which can be placed at any distance. Thus, in the discussion about acute and elective surgical interventions in the pandemic situation because of COVID-19, robot-assisted surgery can demonstrate its importance in a way not previously shown. In that, even complex and intricate oncological surgical interventions can continue to be offered and performed with the highest possible safety for the patient and the surgical team, with the best possible quality.

## Conclusion

Surgery must be able to be offered continuously without loss of quality for both infected and non-infected patients, even in the pandemic situation. The requirements for protective measures no longer only concern the protection of the patient, but increasingly the protection of the staff against infection by aerosols from the patient. Distance to the patient and reduction of the acting persons are current imperatives. In addition, building technology adjustments must be made in the operating theater. 

The conversion of operating theater ventilation to negative pressure operation in accordance with the specifications for isolation rooms with air-lock operation in intensive care units must be implemented.

The choice of the technical operating procedure is not influenced by the COVID-19 situation and should continue to be based on the medical requirements of the illness and the respective expertise of the surgeon. 

Laparoscopic techniques produce aerosols from the capnoperitoneum. Insufflation systems with smoke evacuation and defined CO_2_ supply and removal are preferred. 

Robot-assisted surgery increases the safety aspect for the surgical team, as the decentralized position of the surgeon reduces the number of people needed in the direct surgical field to one. In addition, the globally standardized robotic system available can help to quickly share surgical experience with the system in all regions affected by the pandemic and thus make the virus easy to trace, for the protection of patients and staff alike.

## Key messages

- Surgery under COVID-19 conditions is the new daily routine.
- The change of surgical procedures is necessary to protect patients and staff in the long term.
- Minimally invasive procedures, especially robotics, can be performed with fewer staff in high-risk areas.
- The risk of aerosol entrainment in minimal invasive surgery can be minimized by insufflation systems with flue gas disposal.
- Negative pressure ventilation in the OR tract while maintaining the directional ceiling to floor ventilation (with or without laminar air flow) can be easily and safely produced technically and supports the prompt, routine treatment of COVID-19-affected patients in the OR.

## Notes

### Competing interests

Krüger CM has a consulting mandate with W.O.M. WORLD OF MEDICINE GmbH. The wife of Türler A is an employee of Ethicon Medical GmbH. Kramer A and Riediger H declare that they have no competing interests.
